# Development of pre-implantation genetic testing protocol for monogenic disorders (PGT-M) of Hb H disease

**DOI:** 10.1186/s12864-024-10578-7

**Published:** 2024-07-03

**Authors:** Pannarai Somboonchai, Pimlak Charoenkwan, Sirivipa Piyamongkol, Worashorn Lattiwongsakorn, Tawiwan Pantasri, Wirawit Piyamongkol

**Affiliations:** 1 Department of Obstetrics and Gynaecology, Faculty of Medicine, Chiang Mai University, 110 Intawaroros Road, Sripoom, Mueang, Chiang Mai, 50200 Thailand; 2 Department of Pediatrics, Faculty of Medicine, Chiang Mai University, 110 Intawaroros Road, Sripoom, Mueang, Chiang Mai, 50200 Thailand; 3 Department of Pharmaceutical Sciences, Faculty of Pharmacy, Chiang Mai University, 239 Suthep Road, Suthep, Mueang, Chiang Mai, 50200 Thailand

**Keywords:** Alpha-thalassemia, Hb H disease, Multiplex fluorescent PCR, Preimplantation genetics testing for monogenic disorders (PGT-M)

## Abstract

Hb H disease is the most severe form of α-thalassemia compatible with post-natal life. Compound heterozygous α^0^-thalassemia^− SEA^ deletion/α^+^-thalassemia^− 3.7kb^ deletion is the commonest cause of Hb H disease in Thailand. Preimplantation genetics testing for monogenic disorders (PGT-M) is an alternative for couples at risk of the disorder to begin a pregnancy with a healthy baby. This study aims to develop a novel PCR protocol for PGT-M of Hb H disease^− SEA/−3.7kb^ using multiplex fluorescent PCR. A novel set of primers for α^+^-thalassemia^− 3.7kb^ deletion was developed and tested. The PCR protocol for α^0^-thalassemia^− SEA^ deletion was combined for Hb H disease^− SEA/−3.7kb^ genotyping. The PCR protocols were applied to genomic DNA extracted from subjects with different thalassemia genotypes and on whole genome amplification (WGA) products from clinical PGT-M cycles of the families at risk of Hb Bart’s. The results were compared and discussed. The results showed three PCR products from α^+^-thalassemia^− 3.7kb^ primer set, and three from α^0^thalassemia^SEA^ primer set. The results were consistent with the known thalassemia genotypes. The novel -α^3.7^ primers protocol was also tested on 37 WGA products from clinical PGT-M cycles giving accurate genotyping results and a satisfying amplification efficiency with the ADO rates of 2.7%, 0%, and 0% for *HBA2*, *HBA1*, and internal control fragments, respectively. This novel PCR protocol can precisely distinguish Hb H disease^− SEA/−3.7kb^ from other genotypes. Additionally, this is the first PCR protocol for Hb H disease^− SEA/−3.7kb^ which is optimal for PGT-M.

## Introduction

### Thalassemia

Thalassemia is the most common inherited monogenic disorder, caused by the defect of the globin chain synthesis, resulting in hemoglobin insufficiency, and still highly prevalent in many regions of the world including Southeast Asia, the Indian subcontinent, and Africa. The prevalence of thalassemia has increased in recent years. Approximately 7% of the global population is estimated to be thalassemia carriers. Types of thalassemia are categorized into α-thalassemia and β-thalassemia, identified by α- or β-globin chains where impaired synthesis occurs [1]. The severity of β-thalassemia is determined by the specific defect within the β-globin gene and the consequently affected hemoglobin molecule [2]. Symptoms of β-thalassemia vary in severity and usually include fatigue, weakness, and pale skin. The more severe forms of β-thalassemia can cause bone deformities and an enlarged spleen [3]. The severity of α-thalassemia is determined by the number of mutated genes inherited from the parents, with more mutations leading to more severe forms of the disorder including borderline anemia, moderate anemia, and hydrops fetalis (the most severe anemia), which results in death in utero or shortly after birth [4].

Common deletions of α-thalassemia remove one α-globin gene such as the rightward (-α^3.7^) or leftward (-α^4.2^). Single α-globin gene deletions are found in 15–75% of African Americans, and those in Saudi Arabia, India, Thailand, Papua New Guinea, and Melanesia [5]. These single α-globin gene deletions and other point mutations related to a single α-globin gene are recognized as α^+^-thalassemia mutations. The − 3.7 kb deletion is caused by mis-crossing over in the Z box of the α-globin gene cluster which gives rise to a single α-globin gene (-α^3.7^) on one chromosome and triplicated α-globin genes (ααα^anti 3.7^) on the homologous chromosome. In Thailand, -3.7 kb deletion is the most common α^+^-thalassemia [6–9]. However, since the deleted sequences of the -α^3.7^ allele are identical to those of the wild type (normal α_1_-globin gene) and the α_1_- and α_2_-globin genes are very similar, the development of PCR protocol for − 3.7 kb deletion detection is very difficult.

The deletions removing both α-globin genes on the same chromosome 16 (in cis) or the complete ζ -globin gene cluster are known as the α^0^-thalassemia mutations [10]. The -SEA type of α^0^-thalassemia deletion is found in 14% of people living in the North of Thailand and is the most common cause of Hb H disease and hydrops fetalis syndrome. Compound heterozygous α^0^-thalassemia^− SEA^ deletion/α^+^-thalassemia^− 3.7kb^ deletion (e.g. --^SEA^/-α^−3.7^) give rise to Hb H disease which is the most common type of Hb H disease in Thailand [6–9].

Hb H disease is the most severe form of α-thalassemia compatible with post-natal life and is caused by the presence of α^0^-thalassemia deletion (or α-thalassemia-1) on one chromosome 16 and α^+^-thalassemia deletion (or α-thalassemia-2 i.e. -3.7 kb deletion and -4.2 kb deletion) on the homologous chromosome 16. Symptoms of Hb H disease vary from mild to moderate anemia symptoms, including fatigue, pale skin, and shortness of breath. More severe usually non-deletion Hb H disease forms of the Hb H disease may induce jaundice and an enlarged spleen. In some evidence, pyrexia with some infection can increase Hb H inclusion bodies formation, leading to acute hemolysis crisis and decreased hemoglobin concentration [4, 11].

### Pre-implantation genetics testing for monogenic disorders (PGT-M)

Pre-implantation genetic testing (PGT) is a procedure of embryo genetic analysis for embryo selection to eliminate the risk of inherited conditions [12]. The embryos generated from in-vitro fertilization are biopsied by one of the three sampling methods: day-0 polar body biopsy, day-3 cleavage stage biopsy, or day-5 blastocyst stage biopsy. PGT is categorized into pre-implantation genetic testing for aneuploidy (PGT-A), pre-implantation genetic testing for structural chromosome rearrangement (PGT-SR), and pre-implantation genetic testing for monogenic/single gene disorders (PGT-M) [13]. Several molecular genetic analysis techniques have been developed. Polymerase chain reaction (PCR) is used for PGT-M including Duchenne muscular dystrophy [14], Fragile X syndrome [15], Tay Sachs disease [16], Marfan’s syndrome [17], Myotonic Dystrophy [18], Charcot Marie Tooth type 1 A [19], familial adenomatous polyposis coli (FAPC) [20], Huntington’s chorea [21], severe inherited skin diseases [22], sickle cell anemia [23], spinal muscular atrophy [24], beta-thalassemia [25], congenital adrenal hyperplasia [26], Lesch Nyhan syndrome [27], medium chain acyl CoA dehydrogenase (MCAD) deficiency [28], and α-thalassemia [29]. Data shows no significant difference in pregnancy rates between those from PGT and regular IVF cycles [30].

## Objective

This study aims to develop novel molecular protocol for PGT-M of Hb H disease (--^SEA^/-α^-3.7^) using multiplex fluorescent PCR.

## Materials and methods

### DNA isolation from blood samples and whole genome amplification (WGA) products samples

Subjects with known α-thalassemia genotypes including normal α-globin gene (αα/αα), heterozygous α^+^-thalassemia^− 3.7kb^ deletion (-α^3.7^/αα), homozygous α^+^-thalassemia^− 3.7kb^ deletion (-α^3.7^/-α^3.7^), heterozygous α^0^-thalassemia^− SEA^ deletion (--^SEA^/αα), and Hb H disease^− SEA/−3.7kb^ deletions (-α^3.7^/--^SEA^) were recruited. All participants were 20 years old or over. They were pregnant or non-pregnant women or partners of the couples with known α-thalassemia genotypes from prenatal carrier screening at the antenatal care clinic of Maharaj Nakorn Chiang Mai Hospital, Chiang Mai, Thailand. Blood sample of hemoglobin Bart’s hydrops fetalis i.e. homozygous α^0^-thalassemia^− SEA^ deletion (--^SEA^/--^SEA^) was recruited from umbilical cord blood of a pregnant woman who already had a prenatal diagnosis result. Informed consent was obtained from all participants. Genomic DNAs from 200 µl whole blood samples were isolated using GF-1 Nucleic Acid Extraction Kits (Vivantis, Selangor Darul Ehsan, Malaysia) according to the manufacturer’s protocol. The couples at risk of having the offspring with Hb Bart’s who came through for clinical PGT-M of α^0^-thalassemia^− SEA^ deletion were counseled about the project. Consent was obtained for using the surplus whole genome amplification (WGA) products to test the novel -α^3.7^ PCR protocols. Cell lysis and WGA from blastocyst biopsy were performed as previously described [31]. The project was approved by the Research Ethics Committee of the Faculty of Medicine, Chiang Mai University (study code: OBG-2566-0331).

### -α^3.7^ deletion primers design

**Fig. 1:**
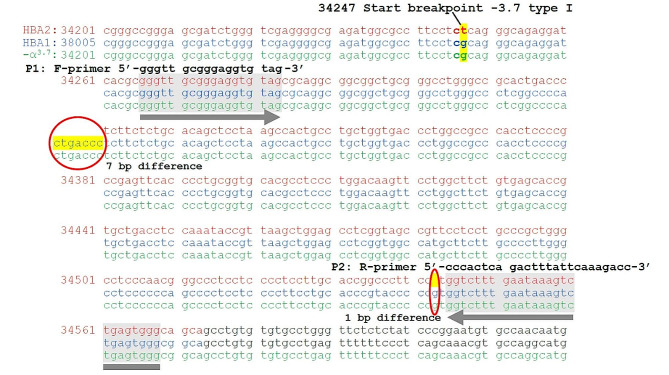
The sequences comparison of the α-globin genes for the differences of wide types i.e. *HbA2* and *HbA1* and the -α^3.7^ deleted allele. The 7-bp and 1-bp deletions of the HBA2 fragment are covered by the -α^3.7^ primer set (5’-GGGTTGCGGGAGGTGTAG-3’ and 5’-CCCACTCAGACTTTATTCAAAGACC-3’) (OMIM: NG_000006.1)

**Fig. 2:**
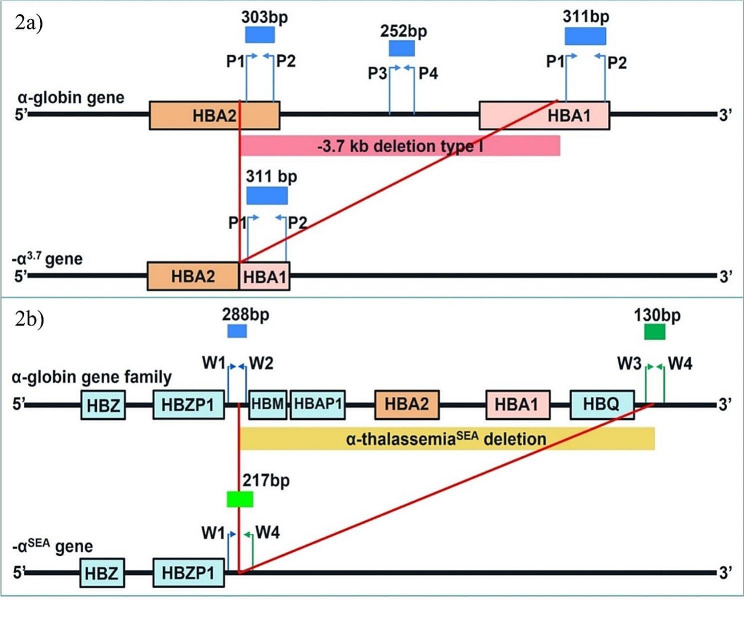
The α-globin genes (OMIM: NG_000006.1) with primers map. **a**) Map of α-globin genes (*HbA2* and *HbA1*), the -α^3.7^ deleted allele and the -α^3.7^ primer set. For normal allele, primers P1 and P2 give rise to 303 bp and 311 bp fragments and primers P3 and P4 give rise to 252 bp fragment. For -α^3.7^ deleted allele, primers P1 and P2 give rise to only 311 bp fragment and primers P3 and P4 do not have any template to amplify (Tables 2 and 1). Map of α-globin genes (*HBM*, *HBAP1*, *HBA2*, *HBA1* and *HBQ*), the -α^SEA^ deleted allele, and the -α^SEA^ primer set [29]. For normal allele, primers W1 and W2 give rise to 288 bp and primers W3 and W4 give rise to 130 bp. For -α^SEA^ deleted allele, primers W1 and W4 give rise to 217 bp fragment (Tables 2 and 1)

α-globin genes situate on the short arm of chromosome 16, containing α_2_ and α_1_ globin genes with very closed similarity in the DNA sequences. The deletion involving α_2_ and α_1_ of -3.7 kb in length causes a part of α_2_ and α_1_ globin genes join to form a hybrid α-globin gene with normal function. Therefore, in case of -3.7 kb deletion, the α_2_ and α_1_ globin genes collapse to become one normal α globin gene. However, the deletion between hemoglobin subunit zeta pseudogene 1 (*HBZP1*) and hemoglobin subunit mu (*HBM*), through the hemoglobin subunit theta (*HBQ*), about 20 kb of nucleotides in length, leads to -SEA deletion, where both α_2_ and α_1_ globin genes were removed. Therefore, Hb H disease^− SEA/−3.7 kb^ deletions (--^SEA^/-α^3.7^) is the combination of -SEA deletion on one allele and − 3.7 kb deletion on the other allele leaving only one functioning α-globin gene to synthesize the α-globin chain. Therefore, primers set for − 3.7 kb deletion was designed to interpret Hb H disease^− SEA/−3.7 kb^ deletions incorporating with the -α^SEA^ primers set which was developed by Piyamongkol W. [29]. DNA sequences of *HBA2* and *HBA1* genes (OMIM: Z84721.1: nucleotide numbers 33,739–34,573 and 37,543–38,385, respectively) were derived from NCBI’s GenBank (https://www.ncbi.nlm.nih.gov/nuccore/NG_000006.1/). It is noted that the α_1_-globin and the -α^3.7^ sequences are identical. The α_2_ and α_1_ globin genes sequences are mostly identical, except for the numbers of the nucleotides whereas the α_1_ globin gene is 8 bp longer than those of the α_2_ globin gene (Fig. 1). Therefore, a novel set of primers was designed to cover the 8 bp difference. In addition, a second set of primers was also designed to amplify the internal control fragment within the breakpoint (Fig. 2).

**Fig. 3:**
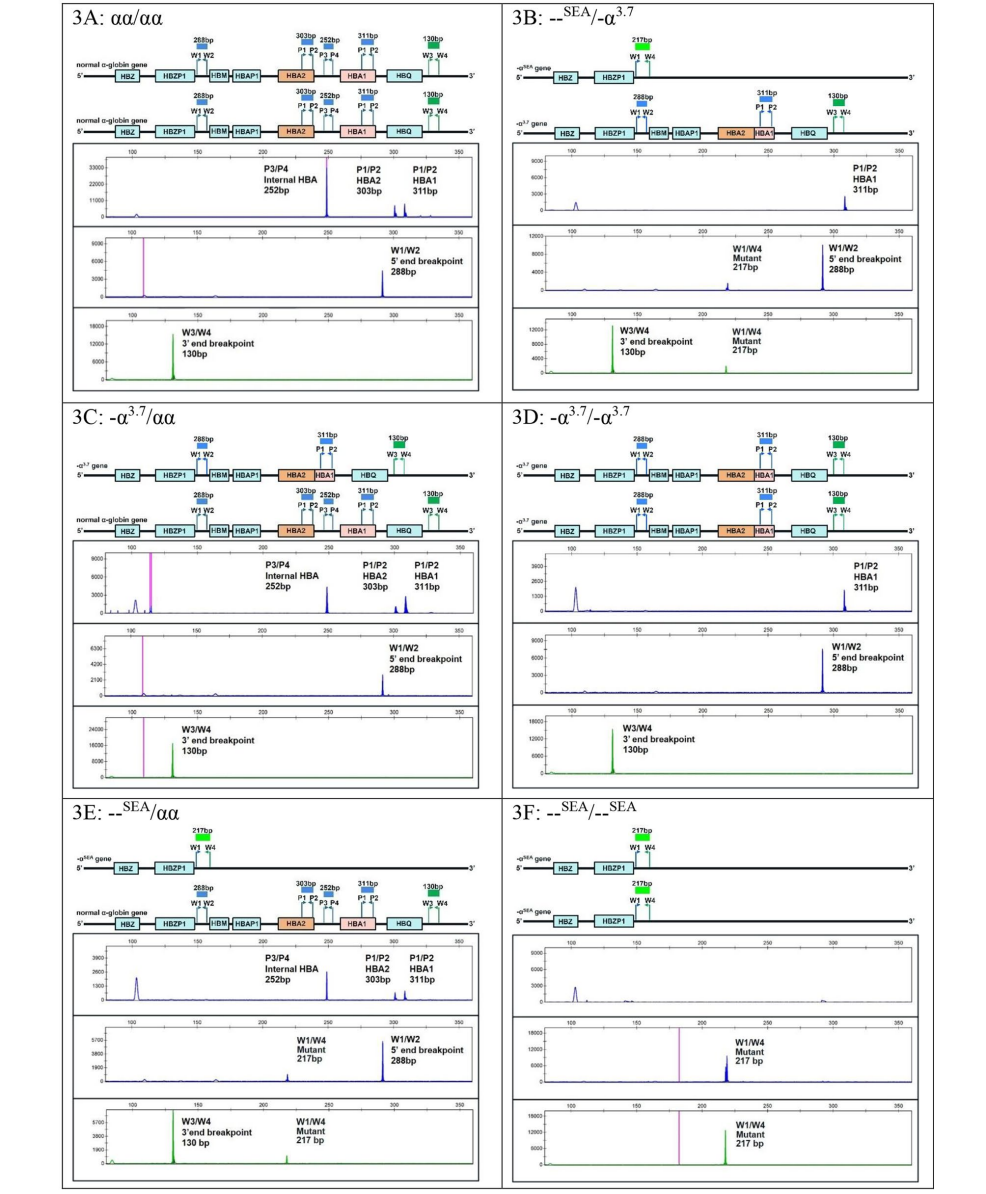
The results of the novel Hb H disease^− SEA/−3.7kb^ genotyping protocol of each α-thalassemia genotype. **A** shows the results of normal α-globin (αα/αα) genotype with positive normal fragments i.e. blue 303 bp *HBA2*, blue 311 bp *HBA1*, blue 252 bp internal control by the -α^3.7^ primer set and normal blue 288 bp 5’ breakpoint and green 130 bp 3’ breakpoint by the -α^SEA^ primer set from the normal αα allele. **B** shows results of the compound heterozygous Hb H disease^− SEA/−3.7kb^ with positive only blue 303 bp *HBA2* fragment from -α^3.7^ allele and normal blue 288 bp 5’ -SEA breakpoint and green 130 bp 3’ -SEA breakpoint and blue/green 217 bp mutant fragment. **C** shows the results of heterozygous − 3.7 kb (-α^3.7^/αα) genotype with positive normal fragments i.e. blue 303 bp *HBA2*, blue 311 bp *HBA1*, blue 252 bp internal control and normal blue 288 bp 5’ -α^SEA^ breakpoint and green 130 bp 3’ -SEA breakpoint. **D** shows results of the homozygous − 3.7 kb (-α^3.7^/-α^3.7^) genotype with positive only blue 311 bp *HBA1* fragments and normal blue 288 bp 5’ -SEA breakpoint and green 130 bp 3’ -SEA breakpoint. **E** shows results of the heterozygous -SEA deletion (--^SEA^/αα) genotype with positive normal fragments i.e. blue 303 bp *HBA2*, blue 311 bp *HBA1*, blue 252 bp internal control and normal blue 288 bp 5’ -SEA breakpoint and green 130 bp 3’ -SEA breakpoint and blue/green 217 bp mutant fragment from the --^SEA^ allele. **F** shows results of the homozygous -SEA deletion (--^SEA^/--^SEA^) genotype with positive only blue/green 217 bp mutant fragment from the --^SEA^ allele

The -α^SEA^ primers set, a combination of W1, W2, W3 and W4 primers as gap-PCR for α^0^-thalassemia^− SEA^ deletion detection was employed. For normal α-globin gene, the W1/W2 primer pair amplifies the products of the 5’ end breakpoint and W3/W4 for 3’ end breakpoint of -SEA deletion giving 288 bp and 130 bp fragments, respectively. For α^0^-thalassemia^− SEA^ deletion, W1/W4 primer amplifies the product of the deleted mutant breakpoint giving a 217 bp fragment (Fig. 3) [29].

### Multiplex fluorescent PCR

0.5 µl of genomic DNAs and WGA products were amplified using a combination of primers. For − 3.7 kb deletion, the -α^3.7^ primers set (P1/P2 and P3/P4 primers) covering *HBA2* and *HBA1* genes. P1 and P4 primers were labeled with the blue fluorescent dye (6’-FAM^®^). For α^0^-thalassemia^− SEA^ deletion, the -α^SEA^ primers set (W1, W2, W3 and W4 primers) [29] covering the SEA deletion breakpoints were employed. W1 and W4 primers were labeled with 6’-FAM^®^ and VIC^®^, respectively. The PCR mixture consisted of 1 pmol/µl of each primer, 1X buffer with MgCl_2_, 0.2 mM dNTPs i.e. dATP, dCTP, dGTP, and dTTP, 0.5 Unit of FastStart Taq and was made up to a total volume of 5 µl with RNase free water. The amplification was performed with the following conditions: pre-denaturation at 95 ºC for 4 min, 35 cycles of 95 ºC for 45 s, 60 ºC for 45 s, and 72 ºC for 60 s. The final extension step was performed at 72 ºC for 10 min. The multiplex fluorescent PCR amplified products were tagged with fluorochromes using labeled primers. This allowed analysis to be performed on an automated laser fluorescent sequencer ABI SeqStudio (Applied Biosystems™, Gibthai Company., Ltd., Bangkok, Thailand).

### Fragment analysis

A mixture of 1 µl of fluorescent PCR products, 10 µl deionized formamide, and 0.1 µl size standard (Genescan^TM^-500 LIZ^®^; Gene Systems Co., Ltd., Bangkok, Thailand) was prepared and denatured at 95 °C for 5 min. The denatured sample was subjected to capillary electrophoresis using Performance Optimized Polymer 7 (POP-7^®^, Gene Systems Co., Ltd., Bangkok, Thailand; 50 s injection time, 15,000 V, 60 °C, 20 min) on an automated DNA sequencer ABI SeqStudio (Applied Biosystems™, Gibthai Company., Ltd., Bangkok, Thailand). The data was analyzed by GeneMapper^®^ software version 4.0 (Gene Systems Co., Ltd., Bangkok, Thailand).

## Results

### Design of novel primers for α-globin genes (-3.7 kb deletion)

DNA sequences of *HBA2* and *HBA1* genes (Z84721.1: nucleotide numbers 33,739–34,573 and 37,543–38,385, respectively) were derived from NCBI’s GenBank (https://www.ncbi.nlm.nih.gov/nuccore/NG_000006.1/). Primers pairs were designed using the Primer-Blast program (https://www.ncbi.nlm.nih.gov/tools/primer-blast/primertool.cgi).

Two new sets of primers for α^+^-thalassemia^− 3.7kb^ deletion analysis, including α-globin gene primers i.e. P1 and P2 primers and internal control α-primers i.e. P3 and P4 primers were designed for -α3.7I: NG_000006.1: g.34247_38050del to detect *HBA2*-*HBA1* and internal control of the wild type α-globin gene. The α-globin gene primer pair (P1/P2) was designed to target *HBA2* and *HBA1* genes [P1: forward-primer 5’GGGTTGCGGGAGGTGTAG-3’ (34,266–34,283, 36,141–36,160 for HBA2 and HBA1, respectively) and P2 reverse-primer 5’-CCCACTCAGACTTTATTC AAAGACC-3’ (34,544–34,568, 38,356–38,380 for *HBA2* and *HBA1*, respectively)]. In the normal α-globin gene, the P1/P2 primers generate the PCR products of 303 and 311 bp for *HBA2* and *HBA1* genes, respectively. For the α^+^-thalassemia (-α 3.7I) gene, the P1/P2 primers give rise to just one PCR product of 311 bp for the *HBA1* gene only since the *HBA2* is deleted. The internal control α-primers pair (P3/P4) was designed to target the internal sequences within the breakpoint between *HBA2* and *HBA1* genes i.e. P3: forward-primer 5’-TGACCTGATGCACTCCTCAA-3’ (36,141–36,160) and P4: reverse-primer 5’-TGTGGTTGGAGAATGGAGGT-3’ (36,373–36,392). The P3/P4 primers generate the PCR products of 252 bp in size. P1 and P3 forward primers were labeled with blue fluorescent dye (6’-FAM^®^). In a normal α-globin genotype sample, the amplification of the P1/P2 primers pair gives rise to 303 bp and 311 bp blue fragments of *HBA2* and *HBA1* genes, respectively. The P3/P4 primers pair amplified a product of 252 bp blue fragment of internal control sequences between the *HBA2* and *HBA1* genes (Fig. 3, A-E).

For the α^0^-thalassemia^− SEA^ deletion, the primers set developed by Piyamongkol W [29] was employed. This primers set uses a combination of W1, W2, W3 and W4 primers as gap PCR for α^0^-thalassemia^− SEA^ deletion detection. In a normal α-globin gene, the W1/W2 primers pair amplifies the 5’ end breakpoint of α^0^-thalassemia^− SEA^ deletion and W3/W4 primers pair amplifies the 3’ end breakpoint. In the α^0^-thalassemia^− SEA^ deletion allele, the W1/W4 primers pair amplify the breakpoint of α^0^-thalassemia^− SEA^ deletion. W1 and W4 forward primers were labeled with blue (6-FAM^®^) and green (VIC^®^) fluorescent dyes, respectively. In a normal α-globin sample, the amplification of W1/W2 and W3/W4 primers pairs gives rise to 288 bp blue and 130 bp green fragments of the normal α-globin gene. In a homozygous α^0^-thalassemia^− SEA^ deletion sample, the W1/W4 primers pair generates a 217 bp blue and green fragment indicating the presence of the deletion (Fig. 3, A-F).

### Multiplex fluorescent PCR results

Single tube multiplex PCR with each of the -α^3.7^ primer sets revealed specific PCR products of the particular genotypes as expected. The samples with normal α-globin gene (αα/αα) and heterozygous − 3.7 kb (-α^3.7^/αα) genotypes showed the PCR products of *HBA2* and *HBA1*, and internal control as 303 bp, 311 bp, and 252 bp, respectively. Only the 311 bp PCR products of HBA1 were detected in the homozygous − 3.7 kb (-α^3.7^/-α^3.7^) and Hb H (--^SEA^/-α^3.7^) samples. No PCR product from the *HBA2* and *HBA1* genes was obtained from the Bart’s hydrops fetalis (--^SEA^/--^SEA^) sample (Fig. 3, A-F).

For -SEA deletion detection, W1, W2, W3 and W4 primers pairs revealed specific PCR products of the particular genotypes as expected. The samples with normal α-globin (αα/αα), the heterozygous − 3.7 kb (-α^3.7^/αα), and the homozygous − 3.7 kb (-α^3.7^/-α^3.7^) genotypes showed the PCR products of normal 5’ breakpoint and 3’ breakpoint of the -SEA deletion of 288 bp and 130 bp, respectively. The heterozygous SEA deletion (--^SEA^/αα) and Hb H (--^SEA^/-α^3.7^) samples showed the PCR products of the normal 5’ breakpoint (288 bp) and 3’ breakpoint (130 bp) of SEA deletion, and the mutant SEA deletion product of 217 bp. The Bart’s hydrops fetalis (--^SEA^/--^SEA^) sample showed only the mutant PCR product of 217 bp (Fig. 3, A-F).

### Testing on WGA products

A total of 37 WGA products samples from clinical PGT-M of α^0^-thalassemia^− SEA^ deletion were tested using the -α^3.7^ primer sets, including 22 heterozygous SEA deletion samples and 15 normal samples. The genotyping results from the -α^3.7^ primer sets aligned with the previously known genotypes. The multiplex fluorescent PCR of the -α^3.7^ primer sets achieved an amplification efficiency of 97.3% for *HBA2*, 100% for *HBA1*, and 100% for internal control fragments, giving the allele drop out (ADO) rates of 2.7%, 0%, and 0% for *HBA1*, *HBA2*, and internal control fragments, respectively.

## Discussion

Hb H disease is the most severe form of α-thalassemia compatible with post-natal life. α-globin genes are unique as there are four functioning genes in an individual, whereas there are two for all other genes. Hb H disease is caused from compound heterozygous α-thalassemia-1/α-thalassemia-2 leading to only one functioning α-globin gene in the patients. The most common mutations causing Hb H disease are the combination of α^0^-thalassemia^− SEA^ deletion (α-thalassemia-1) and α^+^-thalassemia^− 3.7kb^ deletion (α-thalassemia-2), in other words Hb H disease^− SEA/−3.7kb^ [6, 7].

Multiplex PCR is the technique using multiple sets of primers for more than one target sequence to amplify several fragments in a single PCR reaction. This helps in reducing the number of reaction tubes as well as the preparation and analysis time. However, optimization of multiplex PCR is challenging. In the reaction tubes with multiple primer pairs, primers from one pair can interact with primers from the other pair. Additionally, each primer pair needs different ingredients and conditions to optimize the multiplex PCR. Therefore, there is no single optimal ingredient and optimal melting temperature (Tm). When designing primers for multiplex PCR, several factors need to be considered including: the primers length of about 18–25 nucleotides, identical or only 1–2 °C difference melting temperature of the primers, appropriate GC content of the primers (50–55%), and the absence of cross-complementarity to avoid interference within the reaction [32]. Multiplex PCR was first employed for the diagnosis of Duchenne muscular dystrophy in 1988 [33].

Fluorescent PCR (F-PCR) is useful for conditions with low copy number templates by improving the sensitivity and specificity [34]. The primers are tagged with fluorescent dyes causing the amplified PCR products to be labeled with fluorescent dyes. The F-PCR products are then analyzed on capillary electrophoresis with laser intersects and the fluorescent molecules are triggered and emit a signal with a specific wavelength which can be detected by a CCD (charged couple device) sensor and analyzed by computer software. Additionally, the size standards can be run alongside in the same lane with the tested samples in F-PCR. Therefore, size determination is as precise as a single base pair difference [35]. The application of different fluorescent dyes to different primers sets facilitates multiplex PCR. Multiplex F-PCR has been successfully applied in a clinical PGT-M sexing program resulting in a proven pregnancy [36]. Moreover, PGT-M using multiplex F-PCR has been used to identify both α-thalassemia and β-thalassemia [25, 29].

A new set of primers for α^+^-thalassemia^− 3.7kb^ deletion was developed in this study in order to integrate with -α^SEA^ primers set as a PGT-M protocol to diagnose Hb H disease^− SEA/−3.7kb^. The novel -α^3.7^ primers set consists of two pairs of primers. The first pair amplifies 2 unique fragments, one of each within the *HBA2* and *HBA1* genes, and the 303 bp and 311 bp, respectively. The second pair of primers amplifies the third fragment of internal control region within the − 3.7 kb breakpoint, 252 bp. The -α^SEA^ primers set consists of two pairs of primers amplifying wild type breakpoint each of the 5’ and 3’ ends and one deleted breakpoint of the mutant allele as gap PCR. In the optimized PGT-M protocol for Hb H disease^− SEA/−3.7kb^, multiplex fluorescent PCR using 2 sets of primers including -α^3.7^ primers and -α^SEA^ primers to amplify 6 distinct DNA fragments was employed (Fig. 3). The PCR products were analyzed by capillary electrophoresis, which is quick, accurate and sensitive. The primers were labelled with different fluorescent dyes. Fragment analysis software helped in accurately identifying the particular fragments. The results were analyzed for genotyping i.e. heterozygous α^+^-thalassemia^− 3.7kb^ (-α^3.7^/αα), homozygous α^+^-thalassemia^− 3.7kb^ (-α^3.7^/-α^3.7^), heterozygous α^0^-thalassemia^− SEA^ (--^SEA^/αα), homozygous α^0^-thalassemia^− SEA^ (--^SEA^/--^SEA^), and Hb H disease^− SEA/−3.7kb^ (--^SEA^/-α^3.7^) as in the interpretation table (Table 1). The application of the novel -α^3.7^ primers on 37 WGA products from clinical PGT-M cycles gave concordant results to the previously known genotypes and satisfying amplification efficiency with the ADO rates of 2.7%, 0%, and 0% for *HBA2*, *HBA1*, and internal control fragments, respectively. The internal control fragment is the back up result of the *HBA2* fragment.

**Table 1:** The primers details for -α^3.7^ and -α^SEA^ protocols for PGT-M of Hb H disease^− SEA/−3.7kb^

Primer sets	Locations	Primers	Sequences	Labeling Dyes	Products (bp)	References
-α^3.7^ primers	*HBA2* & *HBA1*	P1	5’-GGGTTGCGGGAGGTGTAG-3’	6’FAM	303 (*HBA2*), 311 (*HBA1*)	NG_000006.1
		P2	5’-CCCACTCAGACTTTATTCAAAGACC-3’	-		
	Internal Control *HBA*	P3	5’-TGACCTGATGCACTCCTCAA-3’	6’FAM	252	
		P4	5’-TGTGGTTGGAGAATGGAGGT-3’	-		
-α^SEA^ primers [29]	Normal 5’ Breakpoint	W1	5’-GAAGGAGGGGAGAAGCTGAG-3’	6’FAM	288	NG_000006
		W2	5’-TGTGGAAAAGTTCCCTGAGC-3’	-		
	Normal 3’ Breakpoint	W3	5’-TGCACACCTATGTCCCAGTT-3’	-	130	
		W4	5’-TTGAGACGATGCTTGCTTTG-3’	VIC		
	Deleted Mutant	W1	5’-GAAGGAGGGGAGAAGCTGAG-3’	6’FAM	217	
		W4	5’-TTGAGACGATGCTTGCTTTG-3’	VIC		

The development of primers and PGT-M protocol for − 3.7 kb deletion is sophisticated because the deleted sequences are almost identical to those of the wild type. The sequences of the *HBA2*, HBA1 and deleted − 3.7 kb breakpoint are identical, therefore the concept of gap PCR is not possible. The design of -3.7 kb protocol in this study was based on the amplification of the unique internal control fragments within the *HBA2* and *HBA1* sequences. For this reason, the normal α-globin gene and the heterozygous α^+^-thalassemia^− 3.7kb^ (-α^3.7^/αα) gave the same amplification results using the − 3.7 kb protocol (Table 1) due to the presence of the normal α-globin allele. However, both normal and heterozygous α^+^-thalassemia^− 3.7kb^ are unaffected.

Protocols for α^+^-thalassemia^− 3.7kb^ deletion in the past focused on amplifying across breakpoints. Since the sequences of the *HBA2*, *HBA1* and the deleted α^+^-thalassemia^− 3.7kb^ gene are almost identical, it is very difficult to find unique region around the breakpoints. Therefore, all protocols amplify the fragments of about 2 kb or over which is not appropriate for PGT-M. Primers for α^+^-thalassemia^− 3.7kb^ deletion with restriction fragment length polymorphism (RFLP) by *Apa*I restriction digestion, amplifying -α^3.7^ genes, and internal control of α-globin gene were employed [8]. However, the PCR products were 2,024 bp and 2,274 bp which is not suitable for PGT-M protocol. Chong et al. developed a single-tube multiplex-PCR assay capable of detecting any combination of the 6 common single and double gene deletions including -α^3.7^, - α^4.2^, - α^20.5^, -SEA, -MED, -FIL [37]. However, the PCR products were 2,022 bp for the α^+^-thalassemia^− 3.7kb^ deletion, and 1,800 bp for the *HBA2* gene. Embryo biopsy from PGT-M provides only a few copies number of the DNA templates for the analysis which is not suitable for long length PCR amplification. Appropriate length for PGT-M protocol is 100–500 bp [38]. This study is the first to report the primers sets for the amplification fragments of 130–311 bp in length which is optimal for PGT-M.

## Conclusion

In conclusion, a novel set of primers for multiplex fluorescent PCR was developed and tested to diagnose Hb H disease^− SEA/−3.7kb^. This novel PGD protocol amplifies 6 fragments with different sizes. The -α^3.7^ primers set amplifies three fragments for detecting normal α-thalassemia allele i.e. *HBA2* gene, *HBA1* gene and internal control of the α-globin gene which related with α^+^-thalassemia^− 3.7kb^ deletion. The -α^SEA^ primers set amplifies two fragments for detecting 5’ end breakpoint and 3’ end breakpoint of normal α-globin gene, and one fragment of the deleted breakpoint of the α^0^-thalassemia^− SEA^ deletion. The integration of the results from these two sets of primers helps in the diagnosis of Hb H disease^− SEA/−3.7kb^. The protocol was applied to the DNA samples with known genotypes and the results were concordant to the previously known genotypes. The novel -α^3.7^ primers protocol was also tested on 37 WGA products from clinical PGT-M cycles giving accurate genotyping results and a satisfying amplification efficiency with the ADO rates of 2.7%, 0%, and 0% for *HBA2*, *HBA1*, and internal control fragments, respectively. Additionally, this study reports the first PCR protocol for Hb H disease^− SEA/−3.7kb^ to amplify the fragment length of 130–311 bp which is optimal for PGT-M. Therefore, this multiplex fluorescent PCR protocol can be useful for PGT-M of Hb H disease^− SEA/−3.7kb^.

**Table 2:** Interpretation table of -α^3.7^ and -α^SEA^ protocols for Hb H disease^− SEA/−3.7kb^ genotyping

Primer Sets	Fragments	Genotypes					
		αα/αα	-α^3.7^/αα	-α^3.7^/-α^3.7^	--^SEA^/αα	--^SEA^/--^SEA^ (Hb Bart’s)	--^SEA^/-α^3.7^ (Hb H)
-α^3.7^ primers	*HBA2*/*HBA1*: P1/P2 303/311 bp	**303/311**	**303/311**	**- / 311**	**303/311**	**-**	**- /311**
	Internal *HBA*: P3/P4 252 bp	**+**	**+**	**-**	**+**	**-**	**-**
-α^SEA^ primers [29]	5’ Breakpoint: W1/W2 288 bp	**+**	**+**	**+**	**+**	**-**	**+**
	Deleted Mutant: W1/W4 217 bp	**-**	**-**	**-**	**+**	**+**	**+**
	3’ Breakpoint: W3/W4 130 bp	**+**	**+**	**+**	**+**	**-**	**+**

## Data Availability

No datasets were generated or analysed during the current study.
